# Assessing root canal configuration of permanent mandibular incisors in Indian subjects using CBCT

**DOI:** 10.6026/973206300222138

**Published:** 2026-04-30

**Authors:** Punita Singh, Priyadershini A Rangari, Souryakant Varandani, Mukti Kansal, Shobha Bharti, Ankur Kansal

**Affiliations:** 1 Department of Conservative Dentistry & Endodontics, Patna Medical College & Hospital, Patna, Bihar, India; 2 Department of Dentistry, Nandkumar Singh Chouhan Government Medical College, Khandwa, Madhya Pradesh, India; 3 Department of Pharmacology, Nandkumar Singh Chouhan Government Medical College, Khandwa, Madhya Pradesh, India; 4 Department of Prosthodontics, Yamuna Institute of Dental Sciences and Research, Yamunanagar, Haryana, India; 5 Department of Conservative Dentistry and Endodontics, Buddha Institute of Dental Sciences and Hospital, Patna, Bihar, India; 6 Department of Orthodontics and Dentofacial Orthopedics, Yamuna Institute of Dental Sciences And Research, Yamunanagar, Haryana, India

**Keywords:** Cone beam computed tomography (CBCT), root canal configuration, mandibular incisors, Vertucci's classification

## Abstract

Root canal morphology in mandibular incisors varies widely, complicating endodontic treatment. CBCT offers precise 3D visualization compared with conventional radiography. Therefore, it is of interest to assess root canal configurations in 1,744 permanent mandibular central/lateral incisors from Indian adults. Bifurcated roots occurred in 0.9% overall (0.2% males [n=2/944], 1.0% females [n=8/800]); Vertucci distributions showed no significant right/left or gender differences (p>0.05), through root numbers varied by age group (p< 0.05). Type I configurations predominated bilaterally, consistent with the Indian CBCT series, which reported 58-66% Type I and secondary complex types. This data help by confirming bilateral symmetry and low bifurcation (0.9%) in a large Indian cohort, guiding access preparation and microscopy use for missed canals.

## Background:

The success of endodontic treatment requires careful consideration of the root canal system, root morphology and changes. Endodontic management of the teeth and success is largely governed by the root canal identification, which allows the treatment completion, complete and through root can debridement, including cleaning and shaping and sealing (three-dimensional) and closure of the root canal [1]. Following previous literature, physical changes can occur at varying rates across teeth within each group. The majority of the lower incisors present a single root canal. However, it can be difficult to find root canals in mandibular incisors owing to various anatomical alterations, including tooth apex configuration, lateral root canals and two root canals [2]. The most common reason attributed to endodontic treatment failure in permanent incisors from the mandibular region is untreated disease and/or the presence of an unidentified root canal. It represents anastomosis, external connection, or passageway between two tissue-filled channels [3]. Using CBCT (cone-beam computed tomography), the root apex can be assessed by obtaining high-quality images of the apical outflow tract of the branch, curvature angles, root canal trajectories, resorption, missing canals and abnormalities [4]. In contrast to periapical radiographs, CBCT provides 3-dimensional images of teeth with a comparatively lower radiation exposure. The main cause of root canal deficiency is poor quality and care, due to missing canals or isthmus. Root canal treatment in mandibular incisors can vary by study population and gender [5]. The root of the permanent mandibular incisors is a large oval canal, an accessory root canal, or a lateral root canal that usually branches from a root canal from a medial or coronal third. The reported incidence of missing canals in lower central incisors is 12%, whereas in lower lateral incisors it is 17.4% [6]. This can be attributed to inadequate root canal management in the underlying disease. Anatomic awareness aids proper root canal management, as the tooth's buccolingual width exceeds its mesiodistal width [7]. Previous literature studies have assessed the root canal morphology in the central incisors. In his study, Vertucci assessed 200 lower central and lateral incisors using dye injections and demineralization and reported that 70% and 75% of mandibular central and lateral incisors, respectively, have only a single root canal. Other studies have reported that 79% of mandibular incisors have large canals and apical foramina [8]. CBCT is a valuable and acceptable tool for assessing root canal morphology with high accuracy and eliminating the need for cleaning and dyeing. CBCT also allows assessment of several teeth simultaneously in three dimensions [9]. Therefore, it is of interest to determine and evaluate the root canal configuration in mandibular incisors in Indian subjects using CBCT (cone-beam computed tomography).

## Materials and Methods:

This cross-sectional retrospective study was conducted to determine and evaluate the configuration of the root canal in mandibular incisors in Indian subjects using CBCT (cone-beam computed tomography). The study assessed 1744 CBCT images of the mandibular permanent incisors, including central and lateral incisors, from Indian subjects who presented to the Department of Radiology at the Institute during the defined study period. The study included participants aged 12-60 years who had had completely erupted mandibular incisors with complete root formation, no history of pathologies, crowns or endodontic treatment and had complete root formation as seen on radiographs. The exclusion criteria for the radiographs were the presence of post-restorations or coronal restorations, teeth having immature apices, previously initiated or treated teeth, teeth with periapical radiolucency, root resorption and low-quality images. The study also excluded blurred CBCT images, subjects with a history of orthodontic treatment, developmental disorders, calcifications, root resorption, teeth with open apices and edentulous subjects. The teeth were assessed number of root canals, roots and canal configuration following Vertucci's classification where Type I represented a single canal extending from the pulp chamber to the apex, Type II represented two separate canals taking exit from the pulp chamber and joining to exit as a single canal, Type III depicting one canal leaving the pulp chamber and dividing to two within the root and then joins to exit as one canal, Type IV shows two separate canals leaving the pulp chamber and exit as two separate canals, Type V: One canal leaves the pulp chamber and divides within the body of the root to exit as two separate canals, Type VI: Two separate canals leave the pulp chamber, join within the body of the root and then redivide to exit as two distinct canals, Type VII: One canal leaves the pulp chamber, then divides and rejoins within the body of the root canal and then redivide to exist as two separate canals and Type VIII: Three canals extending from the pulp chamber to the apex. The collected data were entered into Microsoft Excel and their normality was assessed using the Shapiro-Wilk test. Expression of the categorical data was done as frequency and percentage. Intervariable comparison of distribution in different subgroups was done with chi-square and Fisher's test, considering a p-value of > 0.05 as statistically significant. Data analysis was done with SPSS (Statistical Package for the Social Sciences) version 24.0 (IBM Corp., Chicago, IL, USA).

## Results:

Among 1744 subjects assessed in the study, 800 were female and 944 were male. In 800 females, there were 41.5% (n=332), 23.5% (n=188), 21.8% (n=174), 11.8% (n=94), 1% (n=8) and 0.5% (n=4) females in Vertucci's types 1, 2, 3, 4, 5 and 6, respectively. In 944 males, there were 42.4% (n=400), 27.1% (n=256), 22% (n=208), 7.2% (n=68), 0.8% (n=8) and 0.4% (n=2) subjects in Vertucci's types 1, 2, 3, 4, 5 and 6, respectively. This difference was non-significant with p=0.302 (Table 1 - see PDF). On assessing the distribution of mandibular incisors following Vertucci's classification and according to laterality in study subjects, there were 41% (n=358), 25.2% (n=220), 24.1% (n=210), 8.7% (n=76), 0.4% (n=4) and 0.4% (n=4) subjects respectively from Vertucci's type 1, 2, 3, 4, 5 and 6 on the left side. On the right side, there were 42.9% (n=374), 25.7% (n=224), 19.7% (n=172), 9.9% (n=86), 1.4% (n=12) and 0.4% (n=4) subjects respectively from Vertucci's types 1, 2, 3, 4, 5 and 6. The laterality difference was statistically non-significant with p=0.493 (Table 2 - see PDF). The study results showed that for distribution of mandibular incisors following Vertucci's classification and according to teeth type in study subjects, there were 41.9% (n=366), 25.2% (n=220), 21.6% (n=188), 9.4% (n=82), 0.9% (n=8) and 0.9% (n=8) lateral incisors from Vertucci's type 1, 2, 3, 4, 5 and 6 respectively. There were 41.9% 9n=366), 25.7% (n=224), 22.2% (n=194), 9.2% (n=80), 0.9% (n=8) and 0 central incisors from Vertucci's type 1, 2, 3, 4, 5 and 6, respectively. The difference was statistically non-significant with p=0.4075 (Table 3 - see PDF). Concerning the distribution of mandibular incisors following Vertucci's classification and according to age range in study subjects, in age 12-24 years, 25-36 years, 37-48 years and 49-60 years, the majority of subjects were in Vertucci's type 1 followed by type 2 and 3 and type 4 depicting statistical significance with p=0.001 (Table 4 - see PDF).

## Discussion:

The most common reason for incomplete and/or failed root canal treatment in permanent mandibular incisors is attributed to the non- identification of the second canal in these teeth. The reported incidence of the second canal in the permanent lower incisors ranges from 11% to 45%, as reported by Soh and Narayanan in (2013) [10]. CBCT (cone-beam computed tomography) assessment of anatomical structures is accurate and precise [5]. A retrospective CBCT analysis in an Indian subpopulation (Krishnan *et al.* 2024) similarly found 36% of central incisors and 39.5% of lateral incisors with two canals, with bifurcations most frequently occurring in the middle third, reinforcing that mandibular incisors must not be assumed to harbour a single canal alone [11]. CBCT also provides images comparable to the stained and clear methods for assessing the Vertucci distribution type and diagnosing the root canal. It is also considered a non-invasive, effective, reliable and simple method for assessing root canal morphology. CBCT is considered the most accurate technique for identifying additional roots [12]. The main cause of lower teeth treatment is lingual root canal disappearance, leading to necrosis and the release of canal fluids, which reach the apical region via the roots. Hence, a lingual shelf of dentin must be removed and the lingual part of the pulp chamber must be enlarged sufficiently to allow access to the remaining roots of mandibular incisors [13]. In the North Indian population, morphological characteristics indicate that nearly 64% of mandibular incisors have two canals, confirming Vertucci's classification Type I and 36% of mandibular anterior teeth have several secondary root canals, consistent with Type III [6]. Corroborating this, a Kerala-based retrospective CBCT study reported a comparable predominance of Type I configuration in permanent mandibular incisors with Type III as the second most prevalent type, affirming the consistency of Vertucci distribution patterns across Indian subpopulations [14]. Also, previous studies have reported that the formation of two root canals in mandibular incisors is higher in females than in males [15, 16]. However, in the present study, no relationship was seen between root furcation and the gender of study subjects. These findings were consistent with the study by Arsalan *et al.* in (2015) [17], in which the authors retrospectively assessed the morphology of the root canal system in 374 mandibular incisors using CBCT in Turkish subjects from 101 CBCT scans. The authors reported Type 1 (one canal) in 196 teeth and Type 2 (two canals) with varying configurations in 178 teeth. Also, the rate of complex root canal configurations was higher in males than in females. A 2025 retrospective EDDT study on mandibular incisor root canal configurations further confirms that Type I predominates bilaterally without significant right-left variation, and that Type III and Type V represent the principal complex configurations encountered in endodontic practice, consistent with the Vertucci distribution patterns observed across the present study [18]. In contrast, Ghabbani *et al.* (2020), using CBCT on 1,624 mandibular incisors in a Saudi subpopulation, reported that Type III configurations were more prevalent in males (46.9%) while females showed a predominance of Type I (64.8%), suggesting that gender- related differences in canal morphology may be population-specific and less pronounced in Indian subjects. Notably, 98.8% of participants in that study exhibited bilateral symmetry in canal configurations, an observation consistent with the non-significant laterality difference (p=0.493) noted in the present study [19].

## Conclusion:

No gender differences exist in mandibular incisor root canal morphology or root number, though root distribution varies significantly by age group. CBCT reveals rare bifurcations (0.9% overall; 6 right, 4 left teeth with two roots) with bilateral symmetry (p>0.05). Thus, CBCT, dental microscopy and radiographic planning enhance visualization and negotiation of complex canal anatomy.
